# The Response of Microbial Communities to Peatland Drainage and Rewetting. A Review

**DOI:** 10.3389/fmicb.2020.582812

**Published:** 2020-10-29

**Authors:** Ezra Kitson, Nicholle G. A. Bell

**Affiliations:** EaSTCHEM School of Chemistry, University of Edinburgh, Edinburgh, United Kingdom

**Keywords:** fens and bogs, peatland restoration, peatland drainage, microbial communities and soil quality, climate change mitigation, soil metagenomics, peatland rewetting

## Abstract

Peatlands are significant global carbon stores and play an important role in mediating the flux of greenhouse gasses into the atmosphere. During the 20th century substantial areas of northern peatlands were drained to repurpose the land for industrial or agricultural use. Drained peatlands have dysfunctional microbial communities, which can lead to net carbon emissions. Rewetting of drained peatlands is therefore an environmental priority, yet our understanding of the effects of peatland drainage and rewetting on microbial communities is still incomplete. Here we summarize the last decade of research into the response of the wider microbial community, methane-cycling microorganisms, and micro-fauna to drainage and rewetting in fens and bogs in Europe and North America. Emphasis is placed on current research methodologies and their limitations. We propose targets for future work including: accounting for timescale of drainage and rewetting events; better vertical and lateral coverage of samples across a peatland; the integration of proteomic and metabolomic datasets into functional community analysis; the use of RNA sequencing to differentiate the active community from legacy DNA; and further study into the response of the viral and micro-faunal communities to peatland drainage and rewetting. This review should benefit researchers embarking on studies in wetland microbiology and non-microbiologists working on peatland drainage and rewetting in general.

## Introduction

Peatlands cover ∼2.84% of the world’s land area yet account for a significant proportion of terrestrial carbon (Batjes, 1996; Xu et al., 2018). Estimates of the carbon store in northern peatlands have ranged from 500 to 1,055 gigatonnes (Yu, 2012; Nichols and Peteet, 2019). Peatlands act as a carbon sink due to low rates of decomposition leading to the accretion of partially decayed plant matter. Due to their ability to sequester carbon dioxide, peatlands play an important role in mitigating against global climate warming.

Unfortunately in the 20th century peatlands faced anthropogenic alteration on a significant scale, and many were drained in order to repurpose the land for agricultural and industrial use. Based on data from the Greifswald Mire Centre (2015), 527,783 km^2^ of peatland had been drained, most of which occurred in Europe. Analysis of testate amoeba-derived hydrological reconstructions of 31 European peatlands showed that 60% of sites were drier than they have been for the last 600 years (Swindles et al., 2019).

Studies have shown that drained peatlands may act as a net carbon source, as increased oxic conditions promote higher rates of microbial decomposition (Freeman et al., 2001). Accordingly, multiple studies have shown significantly higher CO_2_ emissions from damaged peatlands (Waddington et al., 2002; Knox et al., 2015; Turetsky et al., 2015). Peatlands also face additional threat due to drought induced by climate warming, which risks creating a positive feedback loop between increased peatland CO_2_ emissions and increased warming (Fenner and Freeman, 2011). The evidence for this is equivocal as the effect of increased oxic conditions in drought may be countered by an expansion of vascular plants, in particular shrub vegetation, with high phenolic content which has an inhibitory effect on the heterotrophic respiration of microbes (Wang et al., 2015).

Because of the important role peatlands play in land-atmosphere carbon exchanges, and the numerous other vital ecosystem services that healthy peatlands provide (Marsden and Ebmeier, 2012), there has been considerable effort to restore damaged peatlands in the last few decades. The main restoration methods include the reintroduction of peat forming species, mainly *Sphagnum* mosses, and rewetting by installing dams, refilling drainage ditches and pumping water into the peatland (Thom et al., 2019). Recent analysis suggests that restoration of drained peatlands is the most resource-efficient way of improving soil carbon sequestration (Leifeld and Menichetti, 2018), so we can expect such efforts to increase in scope and scale in the coming decades.

Microorganisms contribute significantly to organic matter transformations within peat soil (Andersen et al., 2013a), and based on the *enzymic-latch theory*, which states that oxygen constraints on the enzyme *polyphenol oxidase* minimize microbial heterotrophic respiration in peat, are the key determinants of the carbon sink or source status of a peatland (Freeman et al., 2001). It is worth noting that oxygen constraints do not solely account for microbial enzyme dynamics in a peatland and additional variables such as pH must be considered (Tahvanainen and Haraguchi, 2013; Kang et al., 2018). Microbial activity in northern peatlands also contributes substantially to global methane (CH_4_) fluxes (Abdalla et al., 2016); net CH_4_ production from near-natural peatlands is maintained by a balance of anaerobic methanogen and aerobic methanotroph populations that are sensitive to changes in water table level. Understanding the response of microbial communities to drainage and rewetting is therefore essential in order to: (a) gauge the success of current restoration efforts; (b) decide on the most appropriate restoration approach for different peat types, and (c) build reliable models of the effect of rewetting on greenhouse gas (GHG) emissions.

The last review into the topic of microbial community responses to peatland drainage and rewetting was by Andersen et al. (2013a). Since then there has been a plethora of new studies published in the field. A search of article abstracts on the Web of Science Core Collection (2020) database using the query AB = [Peat^∗^ AND Microbi^∗^ AND (Rewet^∗^ OR Drain^∗^)] returned 93 results in the period 2013–2020, compared with 74 in the period 1900–2012. Technology driven advances in microbial ecology in the last decade have increased our capacity to probe the functional and genomic content of the peatland microbiome and increased the complexity of the questions that studies can ask. As more researchers around the globe embark on drainage and rewetting peatland microbiology studies a comprehensive review into the literature is therefore desperately needed to orient new researchers in the field. To this end, the aim of this review is to summarize the last 10 years of research into how microbial communities respond to peatland drainage and rewetting and to establish goals and best practices for future research efforts.

**FIGURE 1:**
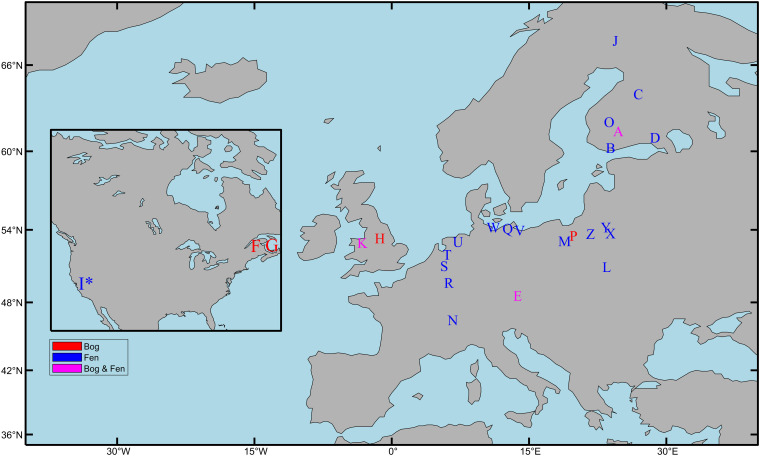
Field locations used by studies investigating the response of microbial communities to peatland drainage and rewetting. See Table 1 for study description (^∗^) = Marsh. Map made using the M_map package in MATLAB (Pawlowicz, 2020).

Peat forms in a variety of wetland ecosystems including bogs, fens, marshes, and swamps. This review will focus on research in minerotrophic (i.e., ground-water fed) fens and ombrotrophic (i.e., rain-water fed) bogs as these peat types are representative of most of the peatlands found in the northern hemisphere (Aselmann and Crutzen, 1989). Fens form in areas where a constant supply of groundwater leads to permanently waterlogged terrain that favors the growth of peat forming species; bogs in comparison only receive water from precipitation and form in upland areas with low rates of evaporation (blanket bogs) or in shallow lakes cut off from ground water supplies (raised bogs). Twenty-one studies across Europe and North America that represent the various aspects of the microbial community response to drainage and rewetting are discussed (Table 1 and Figure 1).

**TABLE 1:** 21 Studies investigating the response of microbial communities to peatland drainage and rewetting.

Location [key] *(peat type(s))*	Microbial analysis method *(depth(s) analyzed)*	Communities Studied	Peatland status	References
Lakkasuo mire, Finland [A] *(Bog; Fen)*	Potential and actual enzyme activities assays. RNA extraction followed by cDNA conversion. PCR-DGGE fingerprint analysis of actinobacteria 16S and fungal 18S RNA genes. Sequencing of DGGE bands. (*Plant litter: 0 cm, 3–5 cm)*	Bacteria; Archaea; Eukaryotes	Near natural; Drained (forestry ditching 1961, controlled ditching 2000).	Strakova et al., 2011
Lakkasuo mire, Finland [A] *(Bog; Fen)*	RNA extraction followed by cDNA conversion. PCR-DGGE fingerprint analysis of actinobacteria 16S and fungal 18S RNA genes. Sequencing of DGGE bands. (*Plant litter: 0 cm, 3–5 cm)*	Fungi; Actinobacteria	Near natural; Drained (forestry ditching 1961, controlled ditching 2000).	Peltoniemi et al., 2012
Six sites in Finland [B; C; D] *(Fen)*	T-RFLP fingerprinting and clone library sequencing of *pmoA* and *mcrA* genes. *(0–7.5 cm, 7.5–15 cm)*	Methanogens; Methanotrophs	Near natural; Rewetted (forestry - drainage ditch blocking ∼1995–1997);	Juottonen et al., 2012
Four sites in Czech Republic [E] *(Bog; Fen)*	PCR-DGGE Fingerprint analysis on *16S RNA* gene. qPCR on *mcrA* gene. *(0–30 cm)*	Methanogens	Near natural; Rewetted (bog only, rewetting by ditch blocking, 2008); Drained (drainage ditches ∼1970s)	Urbanová et al., 2013
Bois-des-Bel bog, Eastern Canada [F] *(Bog)*	Microbial functional diversity and activity calculated using MicroResp system. *(10–20 cm)*	Bacteria; Archaea; Eukaryotes	Near natural; Restored (*Sphagnum* moss transfer and fertilizer addition); Drained (drained and vacuum harvested ∼1970s with exposed bare peat)	Andersen et al., 2013b
Two sites in Eastern Canada [F; G] *(Bog)*	T-RFLP fingerprinting of *16S rRNA* and *mcrA* gene, clone library sequencing of T-RFLP fragments. *(20–30 cm, 30–40 cm)*	Bacteria; Archaea	Near natural (dry bog); Rewetted (ditch blocking ∼1992 [F] and ∼1984 [G] to raise water table); Drained (drained and block-cut actively mined and abandoned ∼1970 sites with exposed bare peat)	Basiliko et al., 2013
Holme Moss, Northern England [H] *(Bog)*	High through-put sequencing of *16S rRNA* gene and *ITS1* region. *(0 – 15 cm)*	Bacteria; Archaea; Fungi	Original vegetation; Restored (managed revegetation and unmanaged revegetation); Drained (drainage ditches with exposed bare peat)	Elliott et al., 2015
Twitchell Island, Western United States [I] *(Marsh)*	High through-put sequencing of *16S rRNA* gene. Whole metagenome sequencing for functional analysis. *(0–12 cm, 12–25 cm)*	Bacteria; Archaea	Rewetted (previously drained by levees in 1869, rewetting using siphon pipe and dams since mid-1990s)	He et al., 2015
Two sites, northern and southern, in Finland. [A; J] *(Fen)*	Microbial respiration and growth rate assay. PLFA based biomass analysis of bacteria and fungi. PCR-DGGE fingerprint analysis of fungal *ITS1* region and sequencing of DGGE bands. qPCR of on ITS and *16S rRNA* genes. *(0–10 cm, 10–20 cm, 20–30 cm, 30–40 cm, 40–50 cm)*	Bacteria; Archaea; Fungi	Near natural; Drained (shallow drainage ditches 2008) (Warming mesocosm experiment)	Peltoniemi et al., 2015
Sumava Mountains, Czech Republic [E] (*Bog; Fen, Swamp)*	High through-put sequencing of *16S rRNA* gene. *(0–30 cm)*	Bacteria; Archaea	Near natural; Drained (drainage ditches ∼1960s)	Urbanová and Bárta, 2016
Migneint, Wales [K] *(Bog)*	Taxonomic identification by eye using a transmitted light microscope. *(0 cm)*	Testate amoebae	Rewetted (ditch blocking/reprofiling ∼2011); Drained (drainage ditches ∼1940s–1970s, burning, afforestation)	Swindles et al., 2016
Two sites, northern and southern, in Finland. [A; J] *(Fen)*	DNA extraction and qPCR of DNA extracts on *mcrA* and *pmoA* genes. RNA extraction and reverse transcription followed by PCR amplification and microarray (*pmoA* gene) or T-RFLP (*mcrA* gene) analysis. Cloning and sequencing of cDNA derived PCR products. *(0–10 cm, 10–20 cm, 20–30 cm, 30–40 cm, 40–50 cm, RNA extraction on 0–20 or 0–40 cm depth)*	Methanogens; Methanotrophs	Near natural; Drained (shallow drainage ditches 2008) (Warming mesocosm experiment)	Peltoniemi et al., 2016
Carbonate – rich fen, Eastern Poland [L] *(Fen)*	Abundance quantification using a variety of lab assays, using DAPI stain, primuline and Lugol’s solution. Taxonomic of protists identification by eye. *(0 cm)*	Bacteria; Heterotrophic flagellates; Ciliates; Testate amoebae	Rewetted (drained in 1960s, rewetted by dams since 2006)	Mieczan and Tarkowska-Kukuryk, 2017
Lakkasuo mire, Finland [A] *(Bog; Fen)*	PLFA analysis of community structure and biomass. *(0–25 cm,25–50 cm, 50–100 cm, bottom)*	Bacteria; Archaea; Fungi	Near natural; Drained (forestry ditching 1961)	Mpamah et al., 2017
Two sites in Wales [K] *(Bog; Fen)*	ARISA fingerprinting, high throughput *16S* and *18S rRNA* gene sequencing. *(5 cm, 20 cm)*	Bacteria; Archaea; Eukaryotes	Near natural (mesocosm drought experiment)	Potter et al., 2017
Linje Mire, Poland and Forbonnet, France [M; N] *(Bog; Fen)*	Flow cytometry, epifluorescence, and light microscopy to estimate bacterial size and abundance. and abundance of microbial consumers and fungal biomass. Phenol oxidase activity assay. *(0 cm)*	Testate amoebae; Ciliates; Rotifers; Nematodes	Near natural (water table manipulation experiment)	Reczuga et al., 2018
Aitoneva, Finland [O] *(Fen)*	T-RFLP fingerprinting using *mcrA* gene and microarray based on semi-nested PCR of *pmoA* gene. qPCR on *mcrA* and *pmoA* gene. *(10–20 cm, 20–30 cm)*	Methanogens; Methanotrophs	Near natural; Rewetted (block cut or milled bogs due to peat removal had become fens, ditch blocking 2008, 1994, 1948).	Putkinen et al., 2018
Mining sites in Poland [P] *(Bog)*	High throughput sequencing of *mcrA* gene. T-RFLP fingerprinting on *pmoA* gene and cloning and sequencing of *mmoX* gene. qPCR on *pmoA*, *mcrA* and *nifH* genes. *(0–10 cm)*	Methanogens; Methanotrophs; Diazotrophs	Near natural; Rewetted (drainage ditches blocked ∼2000); Drained (block cut, surface milled actively mined, and abandoned ∼2004, ∼2009 sites).	Reumer et al., 2018
Two sites in north- eastern Germany [Q] *(Fen)*	High throughput sequencing of *16S rRNA* gene. *(Site 1: 0–5, 5–10, 10–20, 20–30, 30–40, 40–50 cm Site 2: 0–5, 25–30, 50–55 cm)*	Bacteria; Archaea	Rewetted (One site drained in 1970s and rewetted using a dam in 2009, other site drained from 18th century with active pumping in 1970s and rewetted in 2004).	Wen et al., 2018
Linje mire, northern Poland. [M] *(Fen)*	High throughout sequencing of the *18S rRNA* gene. Fluorescence and absorbance assays to measure activity of hydrolase and oxidase enzymes. *(0–8 cm)*	Fungi	Near natural (water table manipulation experiment consisting of wet, ambient, and dry treatments).	Jassey et al., 2018
13 sites in lowland Europe [K; R-Z] *(Fen)*	High throughput sequencing of *16S rRNA* gene. *(0–5, 15–20, 45–50 cm)*	Bacteria; Archaea	Near natural; Rewetted (ditch blocking); Drained (drainage ditches)	Emsens et al., 2020

## Wider Microbial Community

The wider microbial community in near-natural peatlands consists of a diverse assemblage of fungi, bacteria, and archaea (Andersen et al., 2013a). Microbial heterotrophic respiration is the primary contributor (∼70%) to ecosystem respiration from peat soil (Dorrepaal et al., 2009), and so examining wider microbial community structure is critical to understand the impact of drainage and rewetting on land-atmosphere carbon exchanges.

Drainage of fens has caused a decrease in diversity and species richness of the wider microbial community, and a shift toward *Acidobacteria* as the dominant bacterial phylum, non-methanogens as the dominant archaea and saprotrophs as the dominant fungal guild (Urbanová and Bárta, 2016; Jassey et al., 2018; Emsens et al., 2020). In fens, members of the phyla *Proteobacteria* and *Bacteroidetes* have been shown to be particularly sensitive to drought, as well as protists of the phylum *Rhizaria* and ectomycorrhizal fungi (Peltoniemi et al., 2012; Potter et al., 2017). Drainage of fens has been shown to increase microbial activity and modify the active microbial community (Strakova et al., 2011; Mpamah et al., 2017); this is driven by a change in litter type (Peltoniemi et al., 2012) and exhibits a non-linear response to water-table drawdown (Jassey et al., 2018).

Peltoniemi et al. (2015) examined the interaction of warming and drainage status on microbial activity in two boreal fens. There was some interaction between warming and water-level drawdown; both reduced microbial activity in the northern-most fen, although the effects of warming were much more linked to site and depth than the prevailing moisture level. Rewetting has been demonstrated to have a positive impact on the wider fen community structure. In a geographically comprehensive study comparing drained, restored, and near natural sites in thirteen fenlands across Europe, Emsens et al. (2020) showed rewetted fens were more similar to near-natural fens in both their functional and taxonomic diversity. Similarly, He et al. (2015) showed that in a minerotrophic marsh, rewetting was associated with a shift in functional composition; in sites with high water-table there was an increase in *Proteobacteria* and higher gene content for sulfate reduction and denitrification processes. One difference between marsh and fen is the impact of depth on overall community composition: in fenlands, depth shaped community structure more than restoration status (Emsens et al., 2020) whereas the opposite was observed for marshes (He et al., 2015).

The effects of drainage on bog community structuring vary between studies; on a blanket bog in northern England drainage caused a reduction in *16S rRNA* and *ITS1* gene diversity (Elliott et al., 2015) whereas *16S rRNA* gene diversity increased with drainage on a mountain bog in the Czech republic (Urbanová and Bárta, 2016). Other studies using community fingerprinting and *16S rRNA* gene sequencing have reported little to no effect of bog drainage on species diversity and composition (Basiliko et al., 2013; Potter et al., 2017). Peat decomposition rates and overall microbial activity have been shown to decrease in drained bogs (Andersen et al., 2013b; Urbanová and Bárta, 2016) which differs from findings in fens and does not support the theory that peatland drought can cause a positive feedback loop in microbial degradation (Fenner and Freeman, 2011).

On a block-cut (i.e., mined through machine extraction) bog, rewetting was shown to have little impact on overall microbial community structure (Basiliko et al., 2013), and similar to Emsens et al. (2020), depth shaped community structure more than restoration status. Re-vegetation, on the other hand, has a profound influence on microbial structure and activity rates (Andersen et al., 2013b; Elliott et al., 2015). Unsurprisingly, bare peat hosts distinct oligotrophic bacterial and fungal assemblages (Elliott et al., 2015) and multivariate analysis of microbial activity in a Canadian mined bog showed that unvegetated peat had markedly lower and different activity rates than unrestored and restored vegetated peat (Andersen et al., 2013b).

In studies comparing the responses of the wider microbial community in fens and bogs to drainage, fens were shown to be more sensitive (Strakova et al., 2011; Urbanová and Bárta, 2016; Mpamah et al., 2017; Potter et al., 2017). Potter et al. (2017) used a mesocosm experiment to specifically examine the changes in microbial community composition that occur when peatlands experience drought and rewetting. Peat mesocosm cores of 20 cm diameter and 35 cm length were collected from a near-natural bog and a fen in Wales and those in the treatment group were exposed to a gradual lowering of the water table followed by rewetting over a 21-week period. Similar to Basiliko et al. (2013), *16S* and *18S* sequence data did not show a significant effect of drought-rewetting on the overall microbial community composition in any mesocosm. Using linear mixed-effects models, the authors were able to identify operational taxonomic units (OTUs) specifically affected by the interaction between timepoint (since start of the drought manipulation experiment) and treatment. In agreement with Urbanová and Bárta (2016), they found more drought affected OTUs in the fen at 5 cm depth than in any other mesocosm.

## Methanogens and Methanotrophs

Northern peatlands are significant emitters of methane gas (Lai, 2009). While methanogenesis in bogs is largely driven by the hydrogenotrophic (H_2_-CO_2_-dependent) pathway, in fens the acetoclastic (acetate dependent) pathway can be predominant (Horn et al., 2003; Galand et al., 2005). Lowering the water table of peatlands is associated with reduced CH_4_ emissions in a diverse range of peat types (Roulet et al., 1993; Zhang et al., 2019; Planas-Clarke et al., 2020). Drainage increases the exposure of peat soil to oxygen which inhibits methanogenesis and favors methanotrophic oxidation of CH_4_ (Serrano-Silva et al., 2014); rewetting is expected to have the opposite effect and increase long-term CH_4_ emissions from a peatland. This could have significant consequences for future climate warming as CH_4_ has a 100 year global warming potential up to 27 times that of CO_2_ (Boucher et al., 2009). It is therefore essential that we understand the effects of peatland rewetting on the community dynamics of CH_4_ cycling microorganisms.

Drainage of fens was shown to decrease the diversity and abundance of methanogens driven by a change in vegetation cover and quality (Urbanová et al., 2013; Putkinen et al., 2018). The roots of *Carex* and *Eriophorum* species provide acetate for acetoclastic methanogenesis, and due to the presence of aerenchyma (specialized plant tissue that provides oxygen to the submerged root system) can extend into the anoxic layer of peat (Galand et al., 2005; Ström et al., 2005), therefore we expect a reduction in sedge vegetation caused by drainage to limit the abundance and activity acetocolastic methanogens. Furthermore, in addition to increased oxygen saturation, changes to peat soil chemistry caused by drainage such as in increase in phenolic content (Wang et al., 2015; Putkinen et al., 2018), or a decrease in pH (Ye et al., 2012) could inhibit methanogenesis. In particular, a reduction in pH is likely to have a particularly pronounced impact on acetoclastic in fens due to the conversion of acetate into acetic acid at ∼ pH 5.5 (Hao et al., 2012). Contrary to this hypothesis, Putkinen et al. (2018) found that hydrogenotrophic methanogens dominated at all depths in long term rewetted or near natural sites, whereas methanogens capable of acetoclastic production were found only in recently rewetted sites and the bottom layers of the near natural site.

Comparing the effects of 1.5°C experimental warming after a 3-year period on CH_4_ cycling microorganisms in dry and wet regimes in two boreal fens, Peltoniemi et al. (2016) showed that methanogen abundance and CH_4_ production decreased after warming in the wet regimes, but in the drier regimes effects were masked by the significant reduction of methanogen abundance and CH_4_ production due to drainage. Experimental warming studies in a near natural bog [2.25–9°C for 1 year (Wilson et al., 2016)], and freshwater pond mesocosms [4°C for 11 years (Zhu et al., 2020)], have predicted large increases in peatland CH_4_ emissions due to warming. These contrasting findings suggest that in terms of CH_4_ production, fens may be more resilient to warming than bogs and that drainage conceals the effect of warming. However, these studies employed different experimental protocols and factors such as the timescale, degree, and method of warming (i.e., surface warming or deep heating of the core) and the depths analyzed must be accounted for before we can draw reliable conclusions from such comparisons.

The effect of rewetting on the CH_4_-cycling community in fenlands varies between studies. Juottonen et al. (2012) found that CH_4_ emissions were low in restored fens in Finland due to an unresponsiveness of the methanogen community to rewetting, rather than increased CH_4_ oxidation by methanotrophs. Opposite findings were made by Wen et al. (2018) in a study of fens in Germany which showed that methanogens were able to re-establish faster following rewetting than methanotrophs, with a post-rewetting methanogen abundance over two orders of magnitude greater than methanotroph abundance. Putkinen et al. (2018) compared fens that had been rewetted 2, 17, and 63 years ago and demonstrated a recovery of both methanogens and type II methanotrophs that was strongly linked to *Sphagnum* abundance in rewetted oligotrophic fens. Based on these findings it is clear that methanogen recovery is linked to a range of other environmental variables beyond water table, such as vegetation cover, the organic content of soil and pH. As Putkinen et al. (2018) demonstrated, these variables will take time to respond to rewetting, therefore the timescale between microbial analysis and rewetting will have a large effect on the composition of CH_4_ cycling microorganisms that is observed. Juottonen et al. (2012) and Wen et al. (2018) used similar timescales in their analysis (10–12 and 5/10 years post-rewetting, respectively) so it may be that geographic-driven differences in fenland methanogen and methanotroph composition explain the conflicting findings.

In bogs, Reumer et al. (2018) showed that drainage caused by block-cutting compromised the methanogen, methanotroph and diazotroph communities. Rewetting and the reintroduction of *Sphagnum* moss led to a significant increase in the methanogen and diazotroph communities and to a lesser extent the methanotrophs. Drainage favored acetoclastic methanogens but rewetting and *Sphagnum* revegetation was associated with a shift back to hydrogenotrophic methanogens.

In studies comparing the responses of CH_4_ cycling microorganisms in fens and bogs, fens were demonstrated to be more sensitive to environmental perturbation than bogs, with larger shifts in CH_4_ emissions and methanogen community structure (Urbanová et al., 2013; Juottonen, 2020). Using a reciprocal transplant experiment between the two peat types, Juottonen (2020) showed that fen methanogens were severely affected by the shift to the more acidic bog, but the bog microorganisms were not significantly impacted by the reciprocal switch. A cause of this could be disruption of acetoclastic methanogenesis in fens, as low pH prompts dissociation of acetate into acetic acid, but more resilience to increased pH in the hydrogenotrophic pathway in bogs.

## Micro-Fauna

Micro-faunal communities are a fundamental and often understudied component of microbial ecosystems. Testate amoeba are the dominant consumers of other microorganisms in ombrotrophic peatlands and comprise up to 30% of biomass (Mitchell et al., 2003). Through predation they play a significant role in mediating carbon cycling in response to climate warming (Jassey et al., 2015). Furthermore, protozoan grazing can have a significant impact on the composition of soil bacterial communities (Rønn et al., 2002).

Water-table drawdown on a bog and a fen caused a decrease in the mass of larger microbial predators such as testate amoeba, and a decrease in predator-to-prey mass ratio (PPMR) (Reczuga et al., 2018). In line with this finding, rewetting has been shown to increase testate amoebae abundances, particularly the key-indicator genera *Difflugia* and *Centropyxis* in both a bog and a fen (Swindles et al., 2016; Mieczan and Tarkowska-Kukuryk, 2017).

Protozoan grazing has been identified as a key factor in determining overall bacterial abundance in peatlands (Mieczan and Tarkowska-Kukuryk, 2017). In a drought mesocosm experiment, Reczuga et al. (2018), demonstrated that microbial activity is also strongly linked with PPMR; a reduction in PPMR in moderate drought led to an increase in microbial activity, but both PPMR and microbial activity decreased in extreme drought. The authors posited that there is an optimal range of PPMR in which microbial activity is high, below which secondary extinction cascades lead to weaker top-down stimulation of microbial activity. Further *in situ* study of microbial food webs in fens and bogs is needed to asses the generality of this finding.

## Future Directions and Conclusion

The studies discussed here have sampled peatlands in various states of drainage and recovery. Some peatlands have suffered additional destructive alteration such as fire (Swindles et al., 2016), nutrient deposition (Elliott et al., 2015) and mining (Andersen et al., 2013b; Basiliko et al., 2013; Reumer et al., 2018) which will influence the microbial communities in specific ways (Table 1; Andersen et al., 2013a); this can be evidenced by the extreme dysfunctionality in microbial activity and community composition observed in bare peat (Andersen et al., 2013b; Elliott et al., 2015). Additionally, microbial communities vary spatially and temporally within a single peatland (Andersen et al., 2013a); He et al. (2015) showcased major lateral differences in microbial composition across a single peatland, and Urbanová et al. (2013) revealed the presence of methanogens in anoxic microenvironments within surface areas of a peatland.

11 of the 21 studies we reviewed did not account for depth in their experimental design, which may have obscured changes in community composition and microbial activity caused by drainage, given that the main changes are likely to occur below the surface at the oxic-anoxic boundary. Studies on the impact of drainage on enzyme activity have mostly focussed on just the surface layers of peat, which could explain the divergent findings between bogs (Andersen et al., 2013b; Urbanová and Bárta, 2016), and fens (Strakova et al., 2011; Jassey et al., 2018) and thus findings that drainage decreased the enzyme activity in bogs cannot be used to rule of the existence of a positive feedback loop between drought and microbial degradation. The importance of accounting for depth was demonstrated by Mpamah et al. (2017) who found significant effects of drainage on microbial phospholipid-derived fatty acids (PLFA) in the deepest layers of a bog and fen (>100 cm), where no significance was observed in shallower layers.

Inconsistent timescales for analysis following drainage and rewetting represent an additional bottleneck to comparison between studies. While the majority of sites were drained in the 1960’s or 70s, several studies included sites that were drained in the 1940’s (Swindles et al., 2016) or as far back as the 1869 (He et al., 2015). Similarly, the majority of rewetting took place in the mid 2000’s, however, some sites were rewetted just a few years prior to analysis (Urbanová et al., 2013; Swindles et al., 2016), whereas others had been restored several decades earlier (Basiliko et al., 2013; Putkinen et al., 2018). Given that the changes to surface vegetation and soil chemistry which govern the microbial community response may take years to establish after a change to the water table level, it is important to leave appropriate time, we would advise at least 3 years, before studying whether rewetting has been successful. Likewise, in peatlands that have experienced long-term drainage or rewetting, finding appropriate “near natural” control sites, with the same geography and underlying biogeochemistry as study sites, is essential to allow the effects of disturbance to be accurately gauged.

A wide range of techniques to probe microbial abundance and community composition have been used (Table 1). Community fingerprinting techniques such as T-RFLP and ARISA have poorer resolution than high-throughput marker gene sequencing, as they are not able to distinguish between sequences of the same length. The limitations of fingerprinting techniques were highlighted in the discrepancy between the ARISA and *16*/*18S rRNA* datasets in the study by Potter et al. (2017), from which different conclusions could be drawn about the effect of drought and rewetting on microbial community composition.

Several studies have attempted to analyze the functional capacity of the community in response to restoration (Andersen et al., 2013b; He et al., 2015; Emsens et al., 2020). Emsens et al. (2020) used the PICRUSt algorithm, to infer functional gene content from 16S taxonomic data, this technique is severely limited by PCR primer bias and gaps or inaccuracies in the KEGG Orthology database (Langille et al., 2013), as well as the fact that most organisms in the KEGG database were not isolated from peat soil. An alternative is to combine *16S rRNA* gene analysis with functional analysis of shotgun metagenomes as was demonstrated successfully by He et al. (2015), or to perform amplicon sequencing of functional genes of relevance, a technique several studies have already employed to probe the methanogen, methanotroph and diazotroph communities. Moving forward, the most reliable way for future studies to probe microbial functional composition is to integrate sequence data with proteomic and metabolomic datasets (Franzosa et al., 2015).

Only a few studies of the studies analyzed used RNA-based amplicon sequencing (Strakova et al., 2011; Peltoniemi et al., 2012, 2016) and none used metatranscriptomics to examine the active microbial community in response to drainage and rewetting, although these methods have been applied to near-natural peatlands (Lin et al., 2014; Hausmann et al., 2018). One reason for the lack of RNA studies in peat is the difficulty in acquiring high quality data. In addition to the standard difficulties in working with RNA (arising from the ubiquity of RNAse enzymes in the environment and the inherent instability of the RNA molecule), RNA isolation from peat soil is challenging due to the high content of humic substances in peat which are extracted along with RNA using conventional RNA isolation protocols (Wang et al., 2012). These substances interfere with downstream analysis of RNA such as PCR amplification (Tsai and Olson, 1992) and nucleic acid quantification (Zipper et al., 2003) and must therefore be removed prior to or during RNA isolation. Nevertheless in the context of drainage and rewetting accounting for RNA is critical as distortion from legacy DNA may obscure linkages between restoration status, community composition and GHG fluxes. Accounting for RNA is probably most important in shorter timescale studies where recent transformations to microbial community structure will have created a high incidence of legacy DNA that is unrepresentative of the active community.

Considering the components of the microbial community studied, research on the microbial community response to drainage and rewetting in fens and bogs has focussed predominantly on fungi, bacteria, and archaea; few studies have examined the response of micro-faunal communities and none have looked at the roles of viruses. Research on the micro-faunal community has shown the promise of using indicator-taxa abundance as a simple and robust metric of restoration success (Swindles et al., 2016; Mieczan and Tarkowska-Kukuryk, 2017) and the importance of accounting for predation in our understanding of the microbial response to drought (Reczuga et al., 2018). Viruses are the most abundant component of the biosphere, comprising vast metabolic capability, and viral lysis of microbial populations catalyzes biogeochemical cycling by increasing the size of the dissolved and particulate organic matter pool (Suttle, 2005; Dinsdale et al., 2008). In peat soils viruses have been shown to impact carbon degradation and CH_4_ dynamics (Emerson et al., 2018), and are therefore important factors when considering the effect of environmental perturbation on land-atmosphere carbon exchange.

Finally, in terms of geographic coverage of drainage and rewetting microbiology studies in Europe, a comparison of Figure 1 with data from the European Soil Database (Montanarella et al., 2006), highlights Scotland, Ireland, Sweden, and Estonia as countries with high peatland coverage that were not included in any of the studies we examined.

In conclusion, over the last decade we have gained considerable understanding into how peatland microbial communities are affected by drainage and rewetting, but there remain areas for improvement. Continuing this work is critical; a recent model by Günther et al. (2020) analyzed the radiative forcing potential of CO_2_ and CH_4_ emitted from peatlands and concluded that re-wetting of all drained peatlands should be made an instant priority. Although the current evidence shows that rewetting is having a positive impact on restoring near-natural communities, ensuring large-scale restoration efforts are a success will require further research on peatlands in a variety of drained and rewetted states, accounting for spatial (both lateral and vertical) variation of communities in a peatland and the time elapsed since drainage or rewetting occurred. New studies should also aim to employ multi-omic functional analysis, and include RNA sequencing in their methodology, especially in cases where drainage or rewetting has happened recently. Finally, specific attention should be given to probing the response of predators (i.e., microfauna) and pathogens (i.e., viruses) to drainage and rewetting as these components of the community are significant mediators of C cycling in peat soil.

## Author Contributions

EK planned and wrote the manuscript and made the table and figure. NB planned and edited the manuscript. Both authors contributed to the article and approved the submitted version.

## Conflict of Interest

The authors declare that the research was conducted in the absence of any commercial or financial relationships that could be construed as a potential conflict of interest.
